# Measuring Psychological Resilience in Aging: Findings From the Health and Retirement Study and National Longitudinal Study of Adolescent to Adult Health

**DOI:** 10.1093/geroni/igae013

**Published:** 2024-02-13

**Authors:** Miles G Taylor, Tyler Bruefach, Dawn Celeste Carr

**Affiliations:** Pepper Institute on Aging and Public Policy, Florida State University, Tallahassee, Florida, USA; Pepper Institute on Aging and Public Policy, Florida State University, Tallahassee, Florida, USA; Pepper Institute on Aging and Public Policy, Florida State University, Tallahassee, Florida, USA; Claude Pepper Center, Florida State University, Tallahassee, Florida, USA

**Keywords:** Adaptation, Coping, Measurement invariance, Structural equation modeling, Well-being

## Abstract

**Background and Objectives:**

This study aimed to evaluate the measurement properties of 2 emerging psychological resilience (PR) measures constructed for use in large national data sources and to test their reliability across social axes including race/ethnicity, gender, and socioeconomic status.

**Research Design and Methods:**

Using 2006/2008 data, the Simplified Resilience Score and the Add Health Resilience Scale were tested using overall and multigroup measurement models in a structural equation modeling framework.

**Results:**

Both PR measures perform well as reliable, 1-factor latent constructs capturing adaptive capacity at various life stages. Both measures showed measurement consistency across social axes, with specific differences in item measurement across some racial/ethnic groups.

**Discussion and Implications:**

The results indicate these measures represent high quality, consistent measures of PR in nationally representative aging and health data. The availability of reliable, valid measures of PR enables consistent evaluation of resilience in health and aging processes.

**Translational Significance**: We evaluate two reliable and valid psychological resilience measures that are useful for addressing resilience processes in aging and health in large, nationally representative studies on aging. The availability of these measures in publicly available data could be useful in planning and tailoring interventions at multiple life course stages, in different groups, and for specific stressors or adversities as people age. Understanding resources that support resilience in diverse groups and communities can inform policies that maximize adaptability to hardships in older adulthood.

The concept of resilience has gained increasing attention in recent decades as a key factor shaping physical and mental well-being in association with stressful exposures as we age (Smith & Hayslip, 2012; Taylor & Carr, 2021; Zhang & Silverstein, 2022). Although scholars recognize resilience as the ability to recover from stressful situations, there is a lack of consensus on both operationalization and measurement (Windle, 2011). We conceptualize resilience as *an individual’s capacity to navigate adversity through positive adaptation in a manner that protects health and well-being* (Manning, 2013). Based on this definition, the current article evaluates psychological resilience (PR) as measuring an individual’s underlying psychosocial capacity for adaptation should they face stressors or other adversities. Although several validated measures exist to capture PR as an internalized resource (Windle et al., 2011), the vast majority have been validated and utilized in small, clinical, or specialized samples, making generalizations to broader aging populations difficult (Kalaitzaki et al., 2022; Montoya-Williams et al., 2020). To date, no singular or simplified resilience measures exist in population level data used to study aging. Robust and rigorously tested measures of PR in large, representative, longitudinal data sources are needed to understand the processes and outcomes characterizing resilient individuals as they age.

Resilience is also a key factor in understanding health-related inequities that often amplify with age. However, the construct has not been clearly distinguished from the lack of resources and disproportionate stressors often faced by disadvantaged individuals. Resilience is both highlighted and critically addressed among populations of color (Erving et al., 2020; Höltge et al., 2020; Tobin et al., 2022), women (Lowe et al., 2022), sexual/gender minorities (Colpitts & Gahagan, 2016), and other marginalized groups. Although scholarship increasingly emphasizes that resilient adaptation is both necessary and potentially damaging in the face of cumulative structural and interpersonal inequities such as racism, sexism, discrimination, and poverty (Erving et al., 2020; Tobin et al., 2022), scholars have called for emerging work on resilience that is mindful of structural factors shaping health inequities and coping (Mahdiani & Ungar, 2021). PR measurement has been evaluated across key sociological axes such as race/ethnicity, gender, and socioeconomic status, but these studies are primarily based on small clinical samples and are often cross-sectional, marring temporal investigation of short- and long-term outcomes (Wagnild, 2009). We do not advocate for a “one size fits all” or uniformly positive conceptualization of resilient characteristics, processes, or outcomes. However, if we seek to understand differences in the role of PR in health and aging processes, careful and consistent measurement of PR is necessary in longitudinal data representing a diverse aging population. Measurement invariance would suggest measures of PR are consistent in overall construct and item representation across groups. If substantial and systematic measurement variance exists in PR across social axes, researchers run the risk of biased estimates of both descriptive statistics (e.g., means) and predictive effects (e.g., coefficients) in understanding how PR looks or acts across groups. This may lead, for example, to average levels of resilience seeming significantly lower or higher among women or disadvantaged minorities when this actually reflects measurement bias from differences in how groups respond to items on average. Measurement bias may also lead to biased estimates of effects by group leading to stronger observed effects of PR among more educated individuals, for example, in the face of stress or hardship.

The current article addresses these theoretical and methodological gaps by evaluating complementary measures, based on conceptual frameworks of PR, created for use in two different representative national longitudinal studies (the Health and Retirement Study [HRS] and the National Longitudinal Study of Adolescent to Adult Health [Add Health]). These measures are based on an established instrument derived from qualitative analysis (Wagnild & Young, 1993), constructed and validated based on items currently in nationally representative data sources. Using the previously validated Simplified Resilience Score (SRS) and the emerging Add Health Resilience Scale (AHRS), we first discuss and evaluate each measure as representing a single underlying construct of PR emerging from combined individual survey items in publicly available data (construct validity). We then test whether these constructed measures are consistent in capturing PR across social axes including race/ethnicity, gender, and education. Our goal is to highlight novel measurement of PR constructed from existing psychological items in large, representative data sources to promote the study of resilience as a key feature of aging processes and the ways it may work differently across diverse aging populations.

## Theoretical Conceptualizations of Resilience

We conceptualize individuals who are resilient as having “the capacity to navigate adversity through positive adaptation in a manner that protects health and wellbeing” (Manning, 2013). Theoretical frameworks on resilience vary across disciplines (Windle, 2011), but they share core concepts related to (a) the presence of adversity or stressors in human lives and (b) varied experiences of human adaptation and/or functioning after such setbacks (Fletcher & Sarkar, 2013). Systemic and cumulative inequities in life course adversities hinder, rather than promote, resilience (Levine, 2003) suggesting that adequate structural and interpersonal resources are needed to support the development and preservation of PR.

Resilience has been conceptualized and operationalized over the last few decades as (1) a fairly stable internal or psychological resource, (2) a process of coping or adaptation, and (3) the observation of favorable outcomes following adversity or stressors (Smith & Hayslip, 2012).

Our focus here is on the operationalization and measurement of PR as an internalized aptitude for adaptation in the face of challenges (Manning et al., 2016). PR’s focus on adaptation in response to stress or adversity distinguishes this concept and its measurement from other internalized resources, including mastery or optimism, that comprise general beliefs about oneself or the world (Taylor & Carr, 2021; Wagnild & Young, 1993).

Early work on PR focused on *ego-resilience* (Block & Block, 1980) as a function of the personality encompassing fixed traits that promoted adaptability to changing environments. Although research now suggests PR is more developed than fixed, PR is still operationalized as an internalized resource incorporating a “constellation” of characteristics across multiple domains including hardiness, self-reliance, sense of meaning or purpose, balance, humor, and other characteristics that are consistently observed among resilient individuals (Connor & Davidson, 2003; Fletcher & Sarkar, 2013; Wagnild & Young, 1993). Although adaptation and coping are agentic processes, individuals who identify as resilient or experience resilient outcomes often display high levels of positive psychological characteristics across domains that they draw on as needed over the life course (Manning & Bouchard, 2020). Although multiple measures of PR are accepted and used, to our knowledge, no measures have been successfully incorporated into nationally representative studies on aging and health. Our work here highlights measures that have been recently constructed from existing psychosocial items in two representative data sources on aging, providing novel PR measurement based on a widely used measure (Wagnild & Young, 1993) where it has not existed before in such data (Bruefach, 2023; Manning et al., 2016; Taylor & Carr, 2021).

Lacking well-designed and thoroughly tested measures of resilience in longitudinal and representative data limits our ability to understand important health processes that occur in aging. This is particularly important given that PR has both a strong protective association with several self-reported health outcomes among older adults and is observed as a robust buffer of major life course stressors such as combat exposure, chronic condition onset, and widowhood (King et al., 2019; Manning et al., 2016; Taylor et al., 2019). Although PR seems to be relatively stable in middle to later life, research suggests that it can be bolstered or eroded given specific conditions. Understanding the circumstances under which PR can be maximized for individuals prior to stressors or hardships is key to the effectiveness of interventions after such hardships occur.

## Resilience Over the Life Course

Much of the early work on resilient functioning focused on child development, specifically children who had positive outcomes despite adversity (Garmezy et al., 1984; Rutter, 1987). Although personality characteristics fostering resilience, such as optimism, are viewed as more “trait-like,” scholars note that the development and maintenance of PR early and throughout the life course is supported by financial, family, community, and social resources and investments (Levine, 2003). As research on resilience has evolved, the importance of resilience in aging and health processes has gained increased attention (Höltge et al., 2020; Resnick et al., 2023; Ryff et al., 1998). Scholars argue that greater understanding of resilience across adulthood is especially important for how we evaluate and understand health processes in later life (Ryff et al., 1998). As adults move through life they have more experience with assessing, navigating, and adapting to major and minor stressors that could reinforce resilient functioning or hinder it (Manning & Bouchard, 2020). Pathways across early and later adulthood are also marked by changes in resources, with various setbacks and losses becoming more normative as people age. PR, and the individual characteristics within this multidimensional construct, are thought to have different protective effects depending on the type of stressor, environment, and the demographic and social profiles of individuals.

## Health Disparities and Resilience

Resilience has been critiqued by scientists as an oversimplification of complex (or not fully understood) processes, potentially minimizing structural injustices such as racism, sexism, and poverty facing groups who sometimes experience unexpectedly favorable outcomes (Mahdiani & Ungar, 2021) noted as “paradoxes” in health disparities literature (Boen & Hummer, 2019; Louie et al., 2022). Conceptualizing resilience as an unexpected absence of negative outcomes among disadvantaged groups is problematic because it fails to recognize the strengths and protective resources actively fostered among identities and communities often faced with disproportionate injustices as they age. Therefore, it is important to recognize and, when possible, measure resilience resources and strategies across and within groups when examining the presence or unexpected lack of disparities (Erving et al., 2020; Tobin et al., 2022). Increasingly, scholars have shifted away from a deficit focus to highlight internalized and social strengths (Pattillo, 2021) and specific resources such as self-esteem, spirituality, identity, family support, and community embeddedness as mechanisms of robust and resilient coping in diverse populations (Höltge et al., 2020; Tobin et al., 2022). Qualitative work suggests there are common attributes and themes in resilience aptitudes, identities, and processes across gender and race in later life (Manning & Bouchard, 2020), but more work is needed to understand both shared and group-specific aspects of PR in how they may or may not protect health and well-being.

Although many of the accepted measures of PR have been tested and validated across social groups (e.g., Wagnild, 2009), much of this work comes from small samples with varying study designs. In conceptualizing and capturing internal and external sources of resilience in research, PR measurement should function consistently across social axes even if sources and processes of resilience are recognized as culturally and/or environmentally specific (Ungar & Theron, 2020). Any measures of PR deployed in representative data need to be rigorously evaluated across social axes to ensure both the construct of PR and its operationalization do not reflect a narrow conceptualization based on advantaged populations.

## The Present Study

The goal of this study is to present and rigorously test two measures of PR created for use in nationally representative data sources on aging processes based on a long-established measure of resilience (Wagnild & Young, 1993). The SRS was first created for use in the HRS (Manning et al., 2016) and the complementary AHRS was more recently created for use in the Add Health (Bruefach, 2023). The items and scoring of each measure are somewhat unique in line with each specific data source representing different life stages and the existing/available items within each. These measures have been used independently in prior work on aging and health topics. Here, we compare psychometric evaluations of these two measures in capturing PR across adulthood in the United States and test measurement structure and invariance across social axes including race/ethnicity, gender, and socioeconomic status. These findings address the omission of robust measurement of PR in large, nationally representative studies and provide initial evaluation for use of these measures in diverse aging populations.

We address the following research questions:

1. Can a measurement model assuming one latent factor of PR be used to fit existing psychosocial items consistent with resilient functioning in two nationally representative data sources across early and later adulthood?
2. Does the measurement fit of PR vary across social axes in terms of its overall latent structure and relationships with individual psychosocial measures?

## Data and Methods

### Sample

This study draws on two data sources: the HRS and the Add Health (wave 4 public version). These nationally representative data sets are ideal to address the research questions above because both (a) contain psychosocial batteries of individual items consistent with accepted and validated measures of PR (Bruefach, 2023, Manning et al., 2016, Montoya-Williams et al., 2020) and (b) contain information on respondents’ social contexts, including race/ethnicity, gender, and educational attainment. We use HRS and Add Health data collected in the same historical time period (2006–2008) to help account for external and period factors informing respondents’ PR (e.g., the Great Recession). Our broader goal is to leverage nationally representative data from early adulthood into and throughout later life to address how PR shapes health processes and disparities as adults age. Due to sample size limitations in both data sources, analyses were restricted to respondents identifying as non-Hispanic White (“White”), non-Hispanic Black (“Black”), and Hispanic.

#### Health and Retirement Study

The HRS began collecting demographic, housing, household/family, economic, and health data on a nationally representative sample of adults age 51–62 in 1992 and their spouses. Beginning in 1998, this study became a nationally representative study of adults 51+, with core longitudinal data collected biennially, and refresher cohorts added every 6 years (i.e., every third wave). This study uses the RAND longitudinal file, which is a cleaned and easy to use version of commonly used core longitudinal measures data on demographic information, health status, race/ethnicity, gender, educational attainment, and age. Starting in 2006, a questionnaire related to lifestyle and psychosocial factors was added to the HRS. This Leave-Behind Questionnaire (LBQ) was initially distributed to a random half of HRS respondents in 2006, with the random other half completing the survey in 2008. This alternating random sample design continued moving forward. Thus, the entire HRS sample responds took the LBQ over a two-wave (4-year) period. We combined the 2006 and 2008 LBQ data to obtain a full sample of responses on the psychological items included in the SRS (Manning et al., 2016). We included respondents and spouses who were age-eligible for the HRS (i.e., 51+) who answered all 12 items in the LBQ in 2006 or 2008 used for calculation of the SRS. A Full Information Maximum Likelihood (FIML) estimator was used for missingness across other indicators. FIML uses observations with both complete and partial data, allowing researchers to assume that data is missing at random (Bollen, 1989). The final analytic sample includes 14,064 respondents. Because the HRS are pooled data (2006/2008), all analyses are unweighted since survey weights for 2 years cannot be combined (Staff, 2019). Sensitivity analysis by splitting the date by sample/wave year and using the weights provided similar results to what we present here.

#### Add Health

Around 90,000 respondents were initially surveyed in grades 7–12 in 1994–1995 from 80 high schools and 52 “feeder” middle schools. Add Health recruited a representative sample of 20,745 students for a lengthier in-home survey. Follow-up in-home interviews were conducted in 1996 (wave 2), 2001–2002 (wave 3), 2008 (wave 4), and 2016–2018 (wave 5; Harris et al., 2019). In 2008 (wave 4), a number of personality and social psychology items were collected consistent with resilient characteristics and measurement in adulthood. Data on a representative subsample of young adult respondents (age 24–34) participating in wave 4 are publicly available via the Inter-University Consortium for Political and Social Research (*N* = 5,114). Respondents who did not identify as White, Black, or Hispanic/Latinx (*N* = 172) were excluded. To handle missing data among the remaining 4,937 respondents, we employed FIML estimation. Consistent with recommendations on using Add Health data (Chen & Chantala, 2014), and because one survey weight was possible (2008), all presented statistics and analyses with Add Health are weighted.

### Measures

#### Psychological resilience

Psychological resilience measures in both data sets were informed by Wagnild and Young’s Resilience Scale (1993), a well-established, multidimensional psychosocial measure often used in smaller, clinical samples with items emerging from qualitative research among older adults and subsequently validated for use throughout adulthood (Wagnild, 2009). Although both PR measures are based on the Wagnild and Young (1993) scale, the items and scoring of each are somewhat unique based on the available items in each data source. In 2016, the HRS was leveraged to construct the SRS (Manning et al., 2016) now replicated in several studies as a consistent and robust measure of PR in nationally representative data. The SRS has excellent measurement model fit with high reliability across a variety of HRS subsamples, strong construct validity across a number of stressors, health, and well-being outcomes, and predictive efficacy for health outcomes 4–10 times greater than other established psychological resources such as mastery and optimism (King et al., 2019; Manning et al., 2016; Taylor & Carr, 2021; Taylor et al., 2019). The SRS was calculated based on responses to 12 statements in which individuals indicate how strongly they agree/disagree, such as, “I feel it is impossible for me to reach the goals that I would like to strive for,” “So far, I have gotten the important things I want in life,” and “If something can go wrong for me, it will” (see Table 2 for the full list of SRS items). All items were standardized to range between 0 and 1 because items varied in their original ranges. The standardized items were summed with observed values ranging from 2.4 to 12 (α = 0.85 in our analytic sample).

**Table 2.** Psychological Resilience Scale Description and Confirmatory Factor Analyses

Simplified Resilience Score (*N* = 14,064)			Add Health Resilience Scale (*N* = 4,936)		
Item description	*b*/*se*	*r* ^2^	Item description	*b*/*se*	*r* ^2^
1. b_w6do: I can do the things that I want to do.	0.745*** (0.015)	0.253	1. mast2: Other people determine most of what I can and cannot do.^a^	1.016*** (0.062)	0.194
2. b_w4sat: I am satisfied with my life.	0.734*** (0.016)	0.225	2. optim1: I am not easily bothered by things.	0.844*** (0.110)	0.080
3. b_w7hpls: The future seems hopeless to me and I can’t believe that things are changing for the better.^a^	1.067*** (0.019)	0.421	3. optim3: I hardly ever expect things to go my way.^a^	1.707*** (0.109)	0.386
4. b_w10idl: In most ways, my life is close to ideal.	0.743*** (0.017)	0.209	4. optim6: I’m always optimistic about my future.	0.945*** (0.094)	0.132
5. b_w2imp: So far, I have gotten the important things I want in life.	0.621*** (0.016)	0.173	5. mast3: There is little I can do to change the important things in my life.^a^	1.049*** (0.063)	0.200
6. b_w5con: What happens in my life is often beyond my control.^a^	0.970*** (0.014)	0.333	6. mast1: I have little control over the things that happen to me.^a^	1.157*** (0.050)	0.253
7. b_w8disc: When I really want to do something, I usually find a way to succeed at it.	0.567*** (0.013)	0.189	7. optim2: I get stressed out easily.^a^	1.483*** (0.122)	0.224
8. b_w1goal: I feel it is impossible for me to reach the goals that I would like to strive for.^a^	1.127*** (0.020)	0.418	8. selfcon4: In the last 30 days, how often have you felt that difficulties were piling up so high that you could not overcome them?^a^	1.422*** (0.104)	0.206
9. b_w3wrng: If something can go wrong for me, it will.^a^	0.928*** (0.018)	0.297	9. optim4: I rarely count on good things happening to me.^a^	1.784*** (0.110)	0.412
10. b_w12smd: I can do just about anything I set my mind to.	0.638*** (0.015)	0.202	10. selfcon2: I go out of my way to avoid having to deal with problems in my life.^a^	1.170*** (0.077)	0.165
11. b_w13mng: I have a sense of direction and purpose in life.	0.580*** (0.015)	0.168	11. optim7: Overall, I expect more good things to happen than bad.	1.180*** (0.098)	0.231
12. b_w14slv: There is really no way I can solve the problems I have.^a^^,^^b^	1.000*** —	0.441	12. mast4: There is really no way I can solve the problems I have.^a^^,^^b^	1.000*** —	0.236
Model fit			Model fit		
χ^2^ (*df*)	1,797.47 (46)***		χ^2^ (*df*)	156.682 (32)***	
CFI	0.974		CFI	0.981	
TLI	0.962		TLI	0.960	
RMSEA	0.052		RMSEA	0.028	

*Note*s: Item responses range from one to five and are coded so that responses closer to five indicate higher resilience. CFI = Comparative Fit Index; RMSEA = Root Mean Square Error of Approximation; TLI = Tucker–Lewis Index. ***p <.001, **p <.01, *p <.05.

^a^Reverse coded.

^b^Referent item.

Add Health estimates weighted using cross-sectional weights (gswgt4_2). Health and Retirement Study estimates are unweighted.

The AHRS was recently developed (Bruefach, 2023, Bruefach et al., 2021) as a complementary measure to the SRS using psychological items included in wave 4 Add Health questionnaires. Although emerging work introduces a similarly constructed resilience measure based on the Connor Davidson Resilience Scale (Connor & Davidson, 2003; Montoya-Williams et al., 2020), our goal was to create a measure consistent with the previously validated SRS in capturing PR earlier in adulthood. The AHRS includes items such as “Other people determine most of what I can and cannot do,” “I am not easily bothered by things,” and “Overall, I expect more good things to happen than bad” (see Table 2 for full list of AHRS items). All items (12) range from 1 to 5 and were coded so that higher scores indicate higher levels of psychological function (α = 0.79 in our analytic sample). The final measure (presented in Table 1) averages the summed items for each respondent.

**Table 1.** Descriptive Statistics

Variable	Health and Retirement Study (HRS) (*N* = 14,064)			Add Health (*N* = 4,936)		
	Mean/prop (*SD*)	Min	Max	Mean/prop (*SD*)	Min	Max
Psychological resilience^a^	9.16 (1.85)	1.60	12.00	3.74 (0.48)	1.50	5.00
Race/ethnicity						
Non-Hispanic White	0.83	0.00	1.00	0.65	0.00	1.00
Non-Hispanic Black	0.13	0.00	1.00	0.25	0.00	1.00
Hispanic	0.04	0.00	1.00	0.11	0.00	1.00
Sex						
Female	0.58	0.00	1.00	0.54	0.00	1.00
Male	0.42	0.00	1.00	0.46	0.00	1.00
Education						
Bachelor’s degree or higher	0.21	0.00	1.00	0.32	0.00	1.00
Less than a bachelor’s degree	0.79	0.00	1.00	0.68	0.00	1.00
Age	68.91 (9.87)	51.00	104.00	28.88 (1.77)	24.42	33.92

*Notes*: Means derived using Stata 16.0. Add Health estimates weighted using cross-sectional weights (gswgt4_2). HRS estimates are unweighted.

^a^Interitem correlation is 0.85 for the Simplified Resilience Score (HRS) and 0.79 for the Add Health Resilience Scale (AHRS).

#### Demographics

Measurement invariance in the SRS and the AHRS is tested across race/ethnicity, gender, and education. *Race/ethnicity* is measured as mutually exclusive categories of White, Black, and Hispanic (regardless of race). *Gender* is measured using a dichotomous variable of respondents’ sex (female = 1; male = 0). In Add Health, *personal educational attainment* is measured as respondents’ highest education credential, coded 1 for those who earned a bachelor’s degree or higher relative to those who did not earn a bachelor’s degree (0). Because the HRS measures educational attainment using years of education, we created a dichotomous measure of educational attainment that contrasts respondents with 16+ years of education (1) with respondents who reported fewer education years (0). We also conducted sensitivity analyses across *age* (years), using cutoffs based on the median age (younger = 0; older = 1) in each sample, and alternately using standard deviations (*Z*-scores; younger ≤ −1; −1 < middle < 1; older ≥ 1). We found no evidence of measurement variance across age in either measure, so we present findings in Supplementary Tables 1 and 2, which are not discussed in the text.

### Analytic Approach

We assess these two measures for (1) their ability to represent a uniform construct of PR on average in each data source; and (2) their complete and partial measurement invariance across race/ethnicity, gender, and education level. Using Mplus 7.0, we first conduct confirmatory factor analysis (CFA) to examine the associations between SRS and AHRS items and a latent construct representing PR. CFA is an inductive analytic approach used to interrogate relationships between observed and latent variables (Geiser, 2012). The general equation for the measurement model can be expressed as:


X=Λ ξ+δ

Here, a set of observed indicators (*X*) are a function of one or more underlying latent variables (ξ) and a set of factor loading parameters (Λ) corresponding to each indicator (*X*). Measurement error for each indicator is captured by a set of error terms (δ). We specify one latent factor $\xi_{1}$ representing PR for the SRS and AHRS, respectively. To account for patterns in residual errors that stem from other psychological resources, traits, or characteristics, error terms are correlated for items that belong to scales measuring narrower constructs such as mastery, optimism, and self-control. For all models, model fit is assessed using the chi-square test, Comparative Fit Index (CFI), Tucker–Lewis Index (TLI), Root Mean Square Error of Approximation (RMSEA), and differences in these across models. Component fit is determined by the magnitude of standardized factors loadings and *R*^2^ estimates for each item (Geiser, 2012).

We then use multiple-group CFAs to address the second research question, whether PR is consistently measured across social axes. Specifically, we compare the SRS and AHRS across race/ethnicity, gender, and education. Multiple-group CFA is one way to assess *measurement invariance*, or the similarity in a latent construct’s measurement across social groups. When measurement invariance is supported statistically, it suggests that constructs are consistently interpreted conceptually and measures are functionally equivalent across groups. This approach involves comparing model fit indices across nested models that either constrain parameters or allow them to vary across groups (Putnick & Bornstein, 2016). Measurement invariance is typically supported in analyses that identify similarity in (1) latent constructs’ overall structures (*configural invariance*), (2) their relationships to observed indicators (*metric invariance*), and (3) the observed variables’ intercepts across social groups (*full or partial scalar invariance*). Beginning with a model in which the factor loadings and intercepts freely vary (unconstrained), we first examine configural invariance and the similarity in the overall measurement structure. This would suggest that the SRS and AHRS capture one consistent construct of PR across social axes. Configural invariance is generally accepted when alternative fit indices approach adequate model fit (CFI = 0.90; RMSEA = 0.08; Hu & Bentler, 1999).

Next, we compare the freely varying model to one that constrains factor loadings across groups, thereby examining metric invariance. When supported, this indicates that the relationships between the latent factor(s) and observed indicators do not substantially differ. This would suggest that the items comprising the SRS and AHRS are weighted similarly across social axes in comprising one overarching PR construct. Metric invariance is rejected in the presence of a significant chi-square difference test or substantial changes in CFI (−0.01) and other alternate fit indices less sensitive to sample size (Putnick & Bornstein, 2016). Studies suggest that failure to address metric invariance in cross-cultural studies may lead to biased predictive estimates (Chen, 2008).

Finally, we test for both full and partial scalar invariance by comparing the previous model, constraining the factor loadings, to a constrained model where (1) all item intercepts (full invariance) and (2) individual intercepts (partial) are fixed across groups. When supported, full scalar invariance suggests that mean differences in the latent factor(s) account for the entirety of group differences in the shared variance of observed items (Putnick & Bornstein, 2016). Partial scalar invariance in measures is not uncommon, suggesting that some, but not all, item intercepts are equal across groups. Because it is likely there may be differences in some intercepts across groups (reflecting variations across social axes in mean responses over and above the construct), many scholars suggest that measures with partial scalar invariance are still robust in drawing conclusions across groups (see Byrne et al., 1989), especially when differences in item intercepts occur in opposite directions leading to negligible net differences in a summed scale. We reject full and partial scalar invariance when observing both a significant chi-square difference test (see Author Note 1) and at least a 0.01 decline in CFI (change in RMSEA is also provided). In all analyses of Add Health data, appropriate cross-sectional (gswgt4_2) sample weights were used in accordance with Add Health recommendations (Chen & Chantala, 2014). The HRS analyses are all unweighted due to the lack of one appropriate survey weight for two waves of pooled data (2006/2008).

## Results

### Descriptive Statistics

Table 1 shows the average PR score and demographic information for the HRS and Add Health, respectively. The reliability for both resilience scores is high at roughly α = 0.80/0.85. The mean of the SRS is 9.16 and the means of the AHRS is 3.75. In both data sets, more respondents identified as female than male (54% in Add Health; 58% in HRS). In the HRS sample, about 83% of respondents identified as White, 13% as Black, and 4% as Hispanic. Add Health comprises a more racially diverse sample; 65% of respondents identified as White, 25% identified as Black, and 11% identified as Hispanic/Latinx. Respondents in the HRS were less likely to report education levels consistent with earning a bachelor’s degree than respondents in the Add Health (21% vs 32%). The average age for respondents was about 69 years in the HRS and 29 years in Add Health. Notably, variation in age is larger in the HRS sample (*SD* = 9.87) than in the Add Health sample (*SD* = 1.77).

### Confirmatory Factor Analysis

Table 2 shows the factor loadings and explained variance drawn from one-factor measurement models of the SRS and AHRS. Overall, a one-factor model achieves excellent model fit in both data sets. A one-factor model of the SRS shows high CFI (0.974) and TLI (0.962), as well as an RMSEA below 0.06 (0.052). Regarding the AHRS, the CFI (0.981) and TLI (0.960) are both high with a RMSEA (0.028) that is below 0.05. Though there is a notable range in how much variance the latent factors explain in observed SRS and AHRS items, both measurement models suggest strong associations between the latent factors and their respective scale items. The maximum *R*^2^ observed in the HRS model is 44% and the lowest is 17%. The *R*^2^s associated with the AHRS latent factor range between about 41% and about 8%.

### Multiple-Group Analysis

Next, we assess whether PR constructs and measurement (SRS and AHRS) vary across race/ethnicity, gender, and education by testing configural, metric, and scalar invariance. The results strongly support configural and metric invariance across race/ethnicity, gender, and education. Support for scalar invariance is mixed, as some analyses support full scalar invariance and others only support partial scalar invariance. Full scalar invariance was less often achieved in comparisons between Black respondents and their peers, and in AHRS comparisons across gender and education. As we address in the discussion section, this does not seem to foster biased estimates of overall PR in the SRS or AHRS rather than to reflect differences in specific item responses across groups.

#### Race/ethnicity

We first test configural invariance across race/ethnicity, followed by assessments of metric and scalar invariance. Overall, we observed support for configural invariance supporting one-factor models of SRS and AHRS across race/ethnicity. For the SRS, CFIs ranged from 0.976 to 0.953 (Table 3) and the RMSEAs remained low (below 0.06; Table 3 and Supplementary Table 3), suggesting good model fit. Similar results were observed in analyses of the AHRS (CFIs = 0.979–0.964; RMSEAs < 0.04; Table 3 and Supplementary Table 4). These analyses suggest measurement models of these measures follow similar structures across race/ethnicity.

**Table 3.** Testing Measurement Invariance Across Race/Ethnicity, Gender, and Education

Model	Ref model	Simplified Resilience Score (*N* = 14,064)						Add Health Resilience Scale (*N* = 4,936)					
		*X* ^2^ (*df*)	CFI	RMSEA	Δ CFI	Δ RMSEA	Dec	*X* ^2^ (*df*)	CFI	RMSEA	Δ CFI	Δ RMSEA	Dec
Race: B-W													
M1: C	—	1,581.885 (92)***	0.976	0.051	—	—	A	189.857 (64)***	0.979	0.030	—	—	A
M2: M	M1	1,648.104 (103)***	0.976	0.049	0.000	−0.002	A	240.716 (75)***	0.972	0.032	−0.007	0.002	A
M3: S	M2	2,059.298 (114)***	0.969	0.052	−0.007	0.003	A	485.791 (86)***	0.932	0.046	−0.040	0.014	R
M3a: PS	M2	—	—	—	—	—	—	260.742 (81)***	0.969	0.032	−0.003	0.000	A
Race: H-W													
M4: C	—	1,517.624 (92)***	0.977	0.051	—	—	A	197.768 (64)***	0.977	0.034	—	—	A
M5: M	M4	1,565.105 (103)***	0.976	0.049	−0.001	−0.002	A	214.446 (75)***	0.976	0.032	−0.001	−0.002	A
M6: S	M5	1,798.153 (114)***	0.973	0.050	−0.003	0.001	A	252.867 (86)***	0.972	0.032	−0.004	0.000	A
Race: B-H													
M7: C	—	559.101 (92)***	0.953	0.059	—	—	A	142.452 (64)***	0.964	0.037	—	—	A
M8: M	M7	574.701 (103)***	0.953	0.056	0.000	−0.003	A	154.663 (75)***	0.963	0.035	−0.001	−0.002	A
M9: S	M8	700.701 (114)***	0.941	0.060	−0.012	0.004	R	215.139 (86)***	0.940	0.041	−0.023	0.006	R
M9a: PS	M8	602.176 (110)***	0.951	0.056	−0.002	0.000	A	162.790 (81)***	0.962	0.034	−0.001	−0.001	A
Gender													
M10: C	—	1,843.017 (92)***	0.974	0.052	—	—	A	167.437 (64)***	0.985	0.026	—	—	A
M11: M	M10	1,866.045 (103)***	0.974	0.049	0.000	−0.003	A	196.834 (75)***	0.982	0.026	−0.003	0.000	A
M12: S	M11	2,002.755 (114)***	0.972	0.049	−0.002	0.000	A	499.979 (86)***	0.938	0.044	−0.044	0.018	R
M12a: PS	M11	—	—	—	—	—	—	226.531 (82)***	0.978	0.027	−0.004	0.001	A
Education													
M13: C	—	1,827.773 (92)***	0.974	0.052	—	—	A	221.318 (64)***	0.976	0.032	—	—	A
M14: M	M13	1,939.919 (103)***	0.972	0.050	−0.002	−0.002	A	247.155 (75)***	0.974	0.031	−0.002	−0.001	A
M15: S	M14	2,190.055 (114)***	0.968	0.051	−0.004	0.001	A	339.086 (86)***	0.961	0.035	−0.013	0.004	R
M15a: PS	M14	—	—	—	—	—	—	281.385 (84)***	0.970	0.031	−0.004	0.000	A

*Notes:* Invariance abbreviations are C = configural; M = metric; S = scalar; and PS = partial scalar. Race abbreviations are B = Black; H = Hispanic; and W = White. Decisions indicated as Accept (A) and Reject (R), respectively. Subgroups sample sizes, RMSEA confidence intervals, and changes in chi-square values provided in Supplementary Tables 1 and 2. Add Health estimates weighted using cross-sectional weights (gswgt4_2). Health and Retirement Study estimates are unweighted. CFI = Comparative Fit Index; RMSEA = Root Mean Square Error of Approximation.

*p ≤ .05. **p ≤ .01. ***p ≤ .001.

Metric invariance was examined by comparing the unconstrained model (M1) to one constraining factor loadings across groups (M2). Overall, the results support metric invariance across race/ethnicity for both measures. Table 3 shows that although chi-square test statistics suggest declines in model fit, no changes in CFI exceeded the 0.01 threshold (largest decline = 0.007; AHRS) required to reject metric invariance. The results suggest that on average, the associations between PR items and their latent factors do not vary considerably across race/ethnicity.

More substantial changes in model fit were observed in assessments of scalar invariance testing item intercepts across groups. In the HRS, constraining all intercepts across Black and Hispanic older adults led to poorer model fit (Δ CFI = –0.012). In other words, the SRS latent factor does not entirely account for Black–Hispanic differences in each of the observed items. We were able to achieve partial scalar invariance examining each intercept separately. Of the 12 items, 5 items were higher for Hispanic adults and 4 were higher for Black adults, resulting in a small cumulative difference with an extremely small absolute value of 0.184 units of the summed intercepts.

Similarly, the AHRS latent factor did not entirely account for Black–White (Δ CFI = –0.040) and Black–Hispanic (Δ CFI = –0.023) differences in intercepts of all scale items. Table 3 shows that partial scalar invariance across race/ethnicity was supported in models that allowed five item intercepts to vary between Black adults and their White and Hispanic peers. For Black–White comparisons, the cumulative difference in intercepts was 0.209 units of the total score and for Black–Hispanic comparisons, the cumulative difference was 0.845. Thus, the analyses support partial scalar invariance across race/ethnicity, implying that the SRS and AHRS measures account for some, but not all, group differences in the individual psychological items observed.

#### Gender

Table 3 also includes multiple-group analyses of the SRS and AHRS across gender. Results support a one-factor measurement model fits the data well for both women and men. The CFIs exceed 0.970 and the RMSEAs are below 0.06. Comparing the unconstrained models to those constraining factor loadings across gender groups also provides support for metric invariance in both the SRS and AHRS. Though chi-squares were statistically different between the nested models, the changes in CFI are marginal (SRS = 0.000; AHRS = −0.003).

We observed mixed results in assessing scalar invariance by gender in the SRS and AHRS. Although constraining all item intercepts across gender did not substantially affect model fit in the SRS (Δ CFI = −0.002; support), comparing these nested models indicated substantially worse fit in the AHRS (Δ CFI = −0.044; reject). Like analyses of race/ethnicity, this finding implies that the AHRS does not fully explain gender differences in each of the observed items. By allowing 4 of the 12 items to vary across gender in the AHRS, we achieved support for partial scalar invariance (Δ CFI = −0.004). For gender comparisons, the cumulative difference in intercepts was 0.543 absolute units of the total score. Thus, while the AHRS appears to capture PR comparably across gender, a notable difference emerged in the latent construct’s ability to fully explain gender differences in all of the observed psychological items.

#### Education

The final group comparison examined was across educational attainment, or those with and without education levels equivalent to a bachelor’s degree. Like analyses of gender, configural and metric invariance were strongly supported but the SRS and AHRS provide different levels of support for scalar invariance. The high CFIs (SRS = 0.974; AHRS = 0.976) and low RMSEAs (SRS = 0.052; AHRS = 0.032) suggest that a one-factor measurement model similarly fits the data across education levels. Moreover, the associations between SRS (Δ CFI = −0.002) and AHRS (Δ CFI = −0.002) items and the latent constructs did not substantially vary across education level. Constraining the intercepts across educational attainment led to significantly worse model fit in the AHRS, but not in the SRS. The fully constrained model of AHRS items showed a substantial decline in CFI compared to a model freely estimating item intercepts (Δ CFI = −0.013), which was unobserved in the SRS data (Δ CFI = −0.004). Allowing two items to vary freely across educational attainment suggests partial scalar invariance in the AHRS. Results suggest that PR is similarly measured across educational attainment, though as in previous models, not all the shared variance across AHRS items can be attributed to PR.

## Discussion

The goal of this study was to evaluate two new measures of PR created for use in nationally representative data sources of aging U.S. adults in terms of measurement quality and variance across social axes. From our evaluation of two measures based on items from the HRS and Add Health studies, we determined that both are robust measures that do function as single latent factors of PR and function similarly across a range of social axes. Generally speaking, both the SRS from the HRS and the AHRS offer reliable, valid measurement of resilience that can be leveraged in longitudinal studies to understand individual aptitude for adaptation in the face of challenges, and both serve as important tools for better understanding health-related inequalities in aging processes.

More specifically, our findings showed that the SRS met all but one very conservative test of measurement invariance and the AHRS met most tests of measurement invariance. Further, when only partial invariance was met, the detectable cumulative bias was low for descriptive (mean) comparisons across groups. This suggests both measures, based on accepted PR scales in extant clinical research, perform quite well in population-level data on average and across groups. Slight differences in partial scalar invariance were detected across some racial/ethnic groups suggesting mean differences in individual scale items net of PR likely reflect group differences in the ways specific items capture internalized strengths. Although prior resilience research assumes both shared and varied mechanisms in PR across individuals, environments, and stressors (Manning & Bouchard, 2020), emerging research notes the need for scholars to consider how resilient characteristics and processes may be shaped by different experiences, social location, and identity (Taylor et al., 2021; Tobin et al., 2022). Based on our analysis, we find no strong evidence of bias when using the cumulative measures; however, we encourage scholars using these measures to be mindful of group-specific sources of resilience and utilize well controlled regression models and/or test-specific measurement models when generalizing about PR’s effects at the population level.

We also found that the AHRS met criteria of partial rather than full scalar invariance across social axes, which could reflect measurement, age, or cohort differences compared to the SRS. At this time, the Add Health study includes only adults in early and midlife, and it may be that relatively less exposure to a variety of life stressors during this life stage results in more variability in how individuals reflect on their own expected capacity for adaptation. Although the cumulative differences in intercepts were quite small, we encourage researchers comparing group means on the AHRS (as reflecting higher or lower PR across groups) to be somewhat cautious. Group differences in specific item reporting (partial invariance) are observed in other psychological measures in population-representative data that are used frequently by health and health disparities researchers (Goodwill, 2021). Partial scalar invariance has also been observed in multiple established and supported PR measures across contexts and groups (Gucciardi et al., 2011; Liu et al., 2015; van der Meer et al., 2018) Like these other measures, we suggest that when modeling group-specific effects of PR (interactions across race, gender, etc.), scholars should incorporate sensitivity tests allowing freed as well as fixed intercepts on AHRS items showing measurement variance (Putnick & Bornstein, 2016), or alternatively, should conduct sensitivity tests excluding specific items from the AHRS.

We argue that the strengths of this study provide aging researchers with new ways to conceptualize and operationalize reliable and valid resilience measurement in population level data on aging. We also hope to provide insight into the feasibility of using these measures across groups, including suggestions for reducing bias when testing means or effects (i.e., coefficients) across groups. We do note limitations in the measures and our approach, with suggestions for future research. Although both SRS and the AHRS perform well in statistical tests of average and group specific measurement, these measures are based on existing questions drawn from established psychological batteries. Although original measures of PR would be preferable, we argue the SRS and AHRS provide a unique opportunity to study resilience in representative life course data. We also do not examine a myriad of other resilience-related resources which may be somewhat universal (social support) or group-specific (racial identity). Although we focus only on categorical social axes for tests of measurement invariance, we note that many social constructs are not necessarily binary or fixed across time. We also note that physical/mental health and stress are important axes where bias in PR measurement may also reside. Prior research with the SRS and the AHRS suggests that PR may be affected by, and in turn affects, health and well-being (Bruefach, 2023; Taylor & Carr, 2021; Taylor et al., 2021). Future research should carefully consider how stress and health may bias the measurement of PR at different life stages. Finally, our focus was on the measurement of PR, but we encourage future research to investigate the benefits and potential costs/tradeoffs of resilient functioning by incorporating internalized and externalized resources across and among social groups.

In conclusion, our study provides evidence that both the SRS and the AHRS are valid and robust measures for PR for use in research from early adulthood to later life, across social axes. We also outline sensitivities and steps to reduce bias when comparing PR and its effects across groups. These measures have potential to play an important role in advancing scholarship on the role of resilience in shaping health, health responses to stressful life events, and in better understanding aging processes.

## Supplementary Material

igae013_suppl_Supplementary_Tables_S1-S4

## Author Note

1. Rescaled chi-square difference tests (Satorra & Bentler, 2001) were required to account for Add Health’s complex survey design.
