# Supplementary material for: Measuring Psychological Resilience in Aging: Findings From the Health and Retirement Study and National Longitudinal Study of Adolescent to Adult Health
Source: Innov Aging. 2024 Feb 13;8(3):igae013. doi: 10.1093/geroni/igae013 (PMC10972578; doi:10.1093/geroni/igae013)
Supplement: igae013_suppl_Supplementary_Tables_S1-S4 [file igae013_suppl_supplementary_tables_s1-s4.docx]

***Innovation in Aging* Supplementary Material: Miles G. Taylor, Tyler Bruefach, Dawn Celeste Carr. Measuring Psychological Resilience in Aging: Findings from the Health and Retirement Study (HRS) and National Longitudinal Study of Adolescent to Adult Health (Add Health).**

| **Supplementary Table 1. Testing measurement invariance across age in the Simplified Resilience Score (N = 14,064)** | | | | |
| --- | --- | --- | --- | --- |
| **Model** | X^2^ (df) | CFI | RMSEA (90% CI) | Decision |
| **Younger (51-67) & Older (68+)** | |  |  |  |
| Configural Invariance | 1855.701 (92)*** | 0.974 | .052 (.050-.054) | Accept |
| Metric Invariance | 1940.331 (104)*** | 0.973 | .050 (.048-.052) | Accept |
| Full Scalar Invariance | 2325.822 (114)*** | 0.967 | .053 (.051-.054) | Accept |
|  |  |  |  |  |
| **Younger (51-59), Middle (60-74), & Older (75+)** | |  |  |  |
| Configural Invariance | 1895.480 (138)*** | 0.974 | .052 (.050-.054) | Accept |
| Metric Invariance | 2015.110 (160)*** | 0.972 | .050 (.048-.052) | Accept |
| Full Scalar Invariance | 2543.598 (182)*** | 0.964 | .053 (.051-.054) | Accept |
| ***Note:*** chi-square difference tests and CFI used to assess metric/scalar invariance. | | | | |
| 2-Group Comparison – Younger (n=6,447) \| Older (n=7,617) | | | | |
| 3-Group Comparison – Younger (n=3,308) \| Middle (n=6,746) \| Older (n=4,010) | | | | |
| * p ≤ 0.05 \| ** p ≤0.01 \| *** p ≤ 0.001 | | | | |

| **Supplementary Table 2. Testing measurement invariance across age in the Add Health Resilience Scale (N = 4,936)** | | | | |
| --- | --- | --- | --- | --- |
| **Model** | X^2^ (df) | CFI | RMSEA (90% CI) | Decision |
| **Young (24-29) & Old (30-34)** | |  |  |  |
| Configural Invariance | 203.753 (64)*** | 0.979 | .030 (.025-.034) | Accept |
| Metric Invariance | 231.166 (75)*** | 0.976 | .029 (.025-.033) | Accept |
| Full Scalar Invariance | 237.441 (86)*** | 0.977 | .027 (.023-.031) | Accept |
|  |  |  |  |  |
| **Young (24-26), Middle (27- 30), & Old (31-34)** | |  |  |  |
| Configural Invariance | 263.673 (96)*** | 0.976 | .033 (.028-.037) | Accept |
| Metric Invariance | 300.302 (118)*** | 0.974 | .031 (.026-.035) | Accept |
| Full Scalar Invariance | 331.093 (140)*** | 0.973 | .029 (.025-.033) | Accept |
| ***Note:*** chi-square difference tests and CFI used to assess metric/scalar invariance. | | | | |
| 2-Group Comparison –Young (n=2,532) \| Old (n=2,584) | | | | |
| 3-Group Comparison – Young (n=871) \| Middle (n=3,375) \| Old (n=690) | | | | |
| * p ≤ 0.05 \| ** p ≤0.01 \| *** p ≤ 0.001 | | | | |

| **Supplementary Table 3. Measurement invariance across race/ethnicity, gender, and education in the SRS (N = 14,064)** | | | | | | | | |
| --- | --- | --- | --- | --- | --- | --- | --- | --- |
| **Model** | Comparison Model | X^2^ (df) | CFI | RMSEA (90% CI) | $\Delta$X^2^ ($\Delta$df) | $\Delta$CFI | $\Delta$RMSEA | Decision |
| **Black-White** |  |  |  |  |  |  |  |  |
| M1: Configural Invariance | -- | 1581.885 (92)*** | 0.976 | .051 (.048-.053) | -- | -- | -- | Accept |
| M2: Metric Invariance | M1 | 1648.104 (103)*** | 0.976 | .049 (.027-.036) | 66.219 (11)*** | 0.000 | -0.002 | Accept |
| M3: Full Scalar Invariance | M2 | 2059.298 (114)*** | 0.969 | .052 (.050-.054) | 411.194 (11)*** | -0.007 | 0.003 | Accept |
|  |  |  |  |  |  |  |  |  |
| **Hispanic-White** |  |  |  |  |  |  |  |  |
| M4: Configural Invariance | -- | 1517.624 (92)*** | 0.977 | .051 (.049-.053) | -- | -- | -- | Accept |
| M5: Metric Invariance | M4 | 1565.105 (103)*** | 0.976 | .049 (.047-.051) | 47.481 (11)*** | -0.001 | -0.002 | Accept |
| M6: Full Scalar Invariance | M5 | 1798.153 (114)*** | 0.973 | .050 (.048-.052) | 233.048 (11)*** | -0.003 | 0.001 | Accept |
|  |  |  |  |  |  |  |  |  |
| **Black-Hispanic** |  |  |  |  |  |  |  |  |
| M7: Configural Invariance | -- | 559.101 (92)*** | 0.953 | .059 (.055-.064) | -- | -- | -- | Accept |
| M8: Metric Invariance | M7 | 574.701 (103)*** | 0.953 | .056 (.052-.061) | 15.600 (11) | 0.000 | -0.003 | Accept |
| M9: Full Scalar Invariance | M8 | 700.701 (114)*** | 0.941 | .060 (.055-.064) | 126.000 (11)*** | -0.012 | 0.004 | Reject |
| M9a: Partial Scalar Invariance | M8 | 602.176 (110)*** | 0.951 | .056 (.051-.060) | 27.475 (7)*** | -0.002 | 0.000 | Accept |
|  |  |  |  |  |  |  |  |  |
| **Gender** |  |  |  |  |  |  |  |  |
| M10: Configural Invariance | -- | 1843.017 (92)*** | 0.974 | .052(.050-.054) | -- | -- | -- | Accept |
| M11: Metric Invariance | M10 | 1866.045 (103)*** | 0.974 | .049 (.047-.051) | 23.028 (11)* | 0.000 | -0.003 | Accept |
| M12: Full Scalar Invariance | M11 | 2002.755 (114)*** | 0.972 | .049 (.047-.050) | 136.710 (11)*** | -0.002 | 0.000 | Accept |
|  |  |  |  |  |  |  |  |  |
| **Education** |  |  |  |  |  |  |  |  |
| M13: Configural Invariance | -- | 1827.773 (92)*** | 0.974 | .052 (.050-.054) | -- | -- | -- | Accept |
| M14: Metric Invariance | M13 | 1939.919 (103)*** | 0.972 | .050 (.048-.052) | 112.146 (11)*** | -0.002 | -0.002 | Accept |
| M15: Full Scalar Invariance | M14 | 2190.055 (114)*** | 0.968 | .051 (.049-.053) | 250.136 (11)*** | -0.004 | 0.001 | Accept |
| ***Note:*** Subgroups sample sizes are: Black (n=1,768) \| Hispanic (n=1,128) \| White (n=10,870) \| Female (n=8,218) \| Male (n=5,846) \| No College Degree | | | | | | | | |
| (n=10,950) \| College Degree (n=3,114) | | | | | | | | |
| * p ≤ 0.05 \| ** p ≤0.01 \| *** p ≤ 0.001 | | | | | | | | |

| **Supplementary Table 4. Measurement invariance across race/ethnicity, gender, and education in the AHRS (N = 4,936)** | | | | | | | | |
| --- | --- | --- | --- | --- | --- | --- | --- | --- |
| **Model** | Model Comparison | X^2^ (df) | CFI | RMSEA (90% CI) | $\Delta$X^2^ ($\Delta$df) | $\Delta$CFI | $\Delta$RMSEA | Decision |
| **Black-White** |  |  |  |  |  |  |  |  |
| M1: Configural Invariance | -- | 189.857 (64)*** | 0.979 | .030 (.025-.035) | -- | -- | -- | Accept |
| M2: Metric Invariance | M1 | 240.716 (75)*** | 0.972 | .032 (.027-.036) | 50.859 (11)*** | -0.007 | 0.002 | Accept |
| M3: Full Scalar Invariance | M2 | 485.791 (86)*** | 0.932 | .046 (.042-.050) | 245.074 (11)*** | -0.040 | 0.014 | Reject |
| M3a: Partial Scalar Invariance | M2 | 260.742 (81)*** | 0.969 | .032 (.027-.036) | 20.026 (6)** | -0.003 | 0.000 | Accept |
|  |  |  |  |  |  |  |  |  |
| **Hispanic-White** |  |  |  |  |  |  |  |  |
| M4: Configural Invariance | -- | 197.768 (64)*** | 0.977 | .034 (.028-.039) | -- | -- | -- | Accept |
| M5: Metric Invariance | M4 | 214.446 (75)*** | 0.976 | .032 (.027-.037) | 14.484 (11) | -0.001 | -0.002 | Accept |
| M6: Full Scalar Invariance | M5 | 252.867 (86)*** | 0.972 | .032 (.028-.037) | 38.421 (11)*** | -0.004 | 0.000 | Accept |
|  |  |  |  |  |  |  |  |  |
| **Black-Hispanic** |  |  |  |  |  |  |  |  |
| M7: Configural Invariance | -- | 142.452 (64)*** | 0.964 | .037 (.029-.046) | -- | -- | -- | Accept |
| M8: Metric Invariance | M7 | 154.663 (75)*** | 0.963 | .035 (.027-.043) | 12.211 (11) | -0.001 | -0.002 | Accept |
| M9: Full Scalar Invariance | M8 | 215.139 (86)*** | 0.940 | .041 (.035-.048) | 60.476 (11)*** | -0.023 | 0.006 | Reject |
| M9a: Partial Scalar Invariance | M8 | 162.790 (81)*** | 0.962 | .034 (.026-.042) | 8.127 (6) | -0.001 | -0.001 | Accept |
|  |  |  |  |  |  |  |  |  |
| **Gender** |  |  |  |  |  |  |  |  |
| M10: Configural Invariance | -- | 167.437 (64)*** | 0.985 | .026 (.021-.030) | -- | -- | -- | Accept |
| M11: Metric Invariance | M10 | 196.834 (75)*** | 0.982 | .026 (.021-.030) | 29.397(11)*** | -0.003 | 0.000 | Accept |
| M12: Full Scalar Invariance | M11 | 499.979 (86)*** | 0.938 | .044 (.040-.048) | 303.145 (11)*** | -0.044 | 0.018 | Reject |
| M12a: Partial Scalar Invariance | M11 | 226.531 (82)*** | 0.978 | .027 (.023-.031) | 29.697 (11)*** | -0.004 | 0.001 | Accept |
|  |  |  |  |  |  |  |  |  |
| **Education** |  |  |  |  |  |  |  |  |
| M13: Configural Invariance | -- | 221.318 (64)*** | 0.976 | .032 (.027-.036) | -- | -- | -- | Accept |
| M14: Metric Invariance | M13 | 247.155 (75)*** | 0.974 | .031 (.026-.035) | 25.837 (11)** | -0.002 | -0.001 | Accept |
| M15: Full Scalar Invariance | M14 | 339.086 (86)*** | 0.961 | .035 (.031-.038) | 91.931 (11)*** | -0.013 | 0.004 | Reject |
| M15a: Partial Scalar Invariance | M14 | 281.385 (84)*** | 0.970 | .031 (.027-.035) | 34.230 (9)*** | -0.004 | 0.000 | Accept |
| ***Note:*** Changes in chi-square assessed with Satorra-Bentler scaled difference tests. Subgroups sample sizes are: | | | | | | | | |
| Black (n=1,212) \| Hispanic (n=532) \| White (n=3,192) \| Female (n=2,352) \| Male (n=2,760) \| No College Degree (n=3,444) \| College Degree (n=1,668) | | | | | | | | |
| * p ≤ 0.05 \| ** p ≤0.01 \| *** p ≤ 0.001 | | | | | | | | |
